# Current advances in the treatment of myofascial pain syndrome with trigger point injections: A review

**DOI:** 10.1097/MD.0000000000039885

**Published:** 2024-10-04

**Authors:** Nadia Anwar, Xiong Wei, Yuan Jie, Zhao Hongbo, Hao Jin, Zhaoqiong Zhu

**Affiliations:** a Department of Anaesthesiology, Affiliated Hospital of Zunyi Medical University, Zunyi, China; b Department of Rehabilitation Medicine, Zunyi Medical University, Zunyi, China; c Department of Rehabilitation Medicine, Affiliated Hospital of Zunyi Medical University, Zunyi, China.

**Keywords:** efficacy, injection, myofascial pain syndrome, pain, provocative pain points

## Abstract

Myofascial pain syndrome (MPS) caused by trigger points in muscles is a common cause of local or generalized pain, which is clinically common, has a high incidence, and has no specific cure. The most popular and widely used clinical method mainly targets the trigger point for treatment, i.e. trigger point injection (TPI) therapy. The injectable drugs mainly include glucose, saline, local anesthetics, botulinum toxin type A, platelet-rich plasma, platelet-poor plasma, steroid preparations, etc. This method is low cost and less invasive, and early clinical applications have shown good efficacy. In this paper, we have reviewed clinical research progress in treating MPS with TPI therapy.

## 1. Introduction

The original and most commonly accepted definition of Myofascial pain syndrome (MPS) was developed in 1983 in the trigger point manual by Simons and Travell. MPS is chronic regional pain caused by 1 or more myofascial provocation points (also known as trigger points) (MTrP), which manifest as a highly confined and irritable point of tension in the skeletal muscle or fascia that is palpable and produces localized pressure pain when compressed, and induce pain.^[1]^ MPS is 1 of the main causes of skeletal muscle pain and dysfunction, and in severe cases, anxiety, depression, and other negative emotions, further affecting the quality of life of patients. Therefore, safely and effectively addressing this problem has been challenging in pain clinics.

Previous literature shows conventional treatment approaches effective in managing myofascial pain that includes noninvasive treatments such as laser, ultrasound, heat therapy, massage, shockwave, and exercise treatments; and invasive therapies like injection therapy, dry needling (DN) therapy and the acupuncture. Hence, there is no clear known mechanism for MTrP formation; therefore, the most efficient approach for the treatment of MPS is still debatable.

This article briefly reviews the clinical significance of trigger point injection (TPI) therapy in the treatment of myofascial pain. The main objective of this review focuses to explore the current advancement in the efficacy of TPIs on patients with MPS.

Therefore, to investigate the current advances in the treatment of myofascial pain syndrome (MPS) through trigger point injection (TPI), a comprehensive research strategy by encompassing a multifaceted approach has designed; begin by conducting a thorough literature review of recent studies and clinical trials focusing on the efficacy, safety, and optimization of TPI for MPS. Key databases such as PubMed, Cochrane Library, and Google Scholar had been utilized to gather peer-reviewed articles, systematic reviews, and meta-analyses. This study emphasized on recent objective diagnostic tools such as electromyography, ultrasound and infrared thermography and recent innovations, such as the use of novel injectables (e.g., botulinum toxin, lidocaione, glucose, platelets rich and poor plasma injections, corticosteroids) and advancements in imaging-guided techniques that enhance injection precision. Additionally, we also evaluate comparative studies between TPI and other treatment modalities like physical therapy and alternative interventions. This strategy will provide a comprehensive understanding of current advancements and potential future directions in the treatment of MPS with TPI and this review is expected to provide a new strategy and theoretical basis for the prevention and treatment of MPS.

## 2. Etiology and epidemiology

MPS may be caused by trauma, long-term chronic strain, joint dysfunction, stress, or sedentariness, which cause biochemical reactions in muscle tissue and are responsible for the persistent contraction of tight band fibers. One study found that sedentary workers were more likely to develop MPS symptoms than workers who exercised heavily.^[2]^ A study^[3]^ noted that 85% of back pain and 54.6% of chronic head and neck pain are caused by MPS. MTrP is very common and in a study with 224 patients with nonspecific neck pain, all patients were found to have MTrP.^[4]^ In the pain clinic study, approximately 74% to 85% of patients seen cited MTrP as the primary cause of their pain

## 3. Diagnosis of MPS

Although palpation is a key process in the diagnostic process to identify the point of provocation, there is no consensus diagnostic “gold standard” in all national and international studies of MPS to date. Travell and Simons used a descriptive approach, using the finding of painful nodules in tense muscle bands, and the nature and distribution of pain to diagnosis was determined by the finding of pain nodules in tense muscle bands, and the nature and distribution of pain. A 2017 Delphi survey on the diagnosis of MPS proposed 3 criteria as necessary for the diagnosis of MPS: tight bands, hypersensitivity points, and involvement pain, and patients had to meet 2 or more of these criteria to be diagnosed with MPS.^[5]^

Due to the lack of clinical objective outcome measuring tools, there is a barrier to the critical evaluation of the effectiveness of therapeutic interventions. The nonspecific diagnosis and lack of objective clinical measurements for trigger points mean that the evidence for the effectiveness of TPI also remains heterogeneous. Therefore, reliable objective diagnostic tools are needed for the accurate identification of trigger points and quantified evaluation of treatment outcomes. Such measures could be used for understanding the proper diagnosis and natural pattern of trigger points and to determine the underlying mechanism of MPS. The use of objective outcome measure tools could be useful to identify the multi-dimensional physiological, biomechanical, and metabolic changes in the trigger points for patients with MPS.

This study has provided more clarity on the process of diagnosing MPS by clinicians. However, by applying the clinical criteria developed by Travell and Simons, the diagnosis of MPS has historically relied heavily on the clinical history and physical examination performed by an experienced clinician,^[6]^ which should be combined with relatively objective electromyography, infrared thermography, or musculoskeletal ultrasound to improve diagnostic accuracy.

### 3.1. Surface electromyography

Electromyography (EMG) is a precise objective diagnostic tool used to measure electric activity changes in motor units in individuals with myofascial trigger points pain syndrome. Most commonly, invasive elementary (needle) EMG (EMG) is used, although surface EMG (sEMG) recording has become a popular examination for evaluation of the results of conservative and nonconservative treatments in patients with myofascial pain. MTrPs show increased tension in muscles and decreased myoelectric activity, with the decreased activity being attributed to muscle weakness. Irritable muscles can lead to stiffness, spasms, and muscle fiber shortening, which impede relaxation and reduce strength. These changes can be identified by measuring spontaneous electrical activity in surface EMG machines. Wytrazek et al investigated increased spontaneous electrical activity in the trapezius muscle for myofascial pain patients. The results of his study proved the applicability of the sEMG method for the evaluation of the increased amplitude with clinically evaluated MTrPs. sEMG recordings constitute a specific marker for the detection of muscles with trigger points. sEMG is considered a reliable noninvasive screening tool for exploring muscles with trigger points.

### 3.2. Sonoelastography and sonosite ultrasound

With the advancement of ultrasound technology, the quality of scans for soft tissues and musculature has improved considerably. Sonoelastography is a validated method to assess soft tissues’ mechanical properties. Shear-wave elastography (SWE) is a noninvasive imaging technology, sensitive to tissue stiffness and developed to provide quantitative and objective data, such as Young modulus measured in kilopascals (kPa) and local shear wave speed in meters per second (m/s), which has been used for assessing MTrP characteristics. SWE provides a more objective, reliable, and valid method for quantifying muscle stiffness based on acoustically induced shear waves which travel perpendicularly to the compression waves with variate velocities depending on tissue stiffness. Sikdar et al showed that diagnostic ultrasound and Sonoelastography are objectives and reliable diagnostic measures for localizing MTrPs. They found that trigger points appeared as focal, hypoechoic regions of elliptical shape.

Ultrasound diagnostic methods are useful for distinguishing MTrP and their adjacent structures from normal tissues and for providing a quantified description of underlying structural abnormalities related to trigger points, which can be useful for determining the pathogenesis and pathophysiology of painful MTrP. As ultrasound imaging is a noninvasive approach with a quick scanning time, without radiations, and can visualize target structures and needles in real-time, it has become an ideal tool for guidance procedures as well as for accurate diagnosis and management of pain conditions.

### 3.3. Infrared thermography

Infrared thermography is a useful tool to objectively quantify functional abnormalities leading to skin temperature changes. It is a noncontact and noninvasive painless method based upon infrared radiation emission from the body with a temperature greater than zero. It enables body surface temperature distribution and changes processing. The analysis of skin temperature distribution may indicate subcutaneous blood perfusion and microcirculatory changes, which correlate with local or global physiological conditions. AlmirVieira Dibai-Filho established an analysis on infrared thermography usage for assessing MTrPs and found some useful impacts of skin temperature changes on the presence of trigger points. As MTrP causes autonomic and metabolic muscle activities, infrared thermography provides the possibility of assessing these MTrP metabolic changes. It is a fast, real-time, reliable, noninvasive, and nonionizing method that can be used as a quick diagnostic method mainly as the initial diagnosis of conditions associated with tissue temperature alterations.

## 4. Treatment of MPS

According to research findings over the years, although the most effective treatment is still not conclusive, considerable efficacy has been achieved using different approaches to myofascial pain, mainly divided into invasive and noninvasive therapies, including physiotherapy, medication internalization, exercise therapy, and provocative pain spot injections, physical therapy strategies are often limited by the lack of standardization of techniques and experienced physicians and facilities; long-term adverse reactions to medication and interactions with other medications are not uncommon, especially in older patients with other chronic conditions.^[7]^ In most cases, medications can only achieve temporary and partial symptom relief without reducing the frequency or intensity of recurrence; exercise therapy requires patients to overcome pain and other symptoms and requires good compliance to be effective. Conventional treatment methods, primarily involving physical therapy, aim to alleviate pain, restore function, and prevent recurrence. The key methods include exercise therapy, DN, and various modalities.

### 4.1. Exercise therapy

Exercise therapy for MPS focuses on improving muscle strength, flexibility, and endurance, as well as reducing muscle tension and preventing future episodes of pain.

#### 4.1.1. Stretching exercises

These exercises aim to relieve muscle tightness and reduce the severity of trigger points. Static stretching, such as the use of the “stretch-and-hold” technique, is commonly employed to lengthen affected muscles and improve their range of motion.

#### 4.1.2. Strengthening exercises

Targeted strengthening exercises help address muscle imbalances and provide better support to the affected muscles. Exercises such as resistance training can strengthen weakened muscles, thereby reducing the stress on trigger points and improving overall muscle function

#### 4.1.3. Postural training

Since poor posture can contribute to MPS, exercises aimed at improving posture and body mechanics are often included. Techniques such as ergonomic training and core stabilization exercises help in correcting alignment issues that exacerbate muscle pain

### 4.2. Dry needling

Dry needling (DN) targets trigger points directly with fine needles to relieve pain and improve function. The process involves inserting a thin needle into the trigger point without the use of any injected substances. The goal is to elicit a local twitch response which can reduce muscle hypertonicity and decrease pain. DN is thought to work by disrupting the contracted muscle fibers, promoting local muscle relaxation, and improving blood flow. This can lead to a reduction in the biochemical and neuromuscular activity that maintains the trigger point clinical studies suggest that DN can be effective in reducing pain and improving range of motion in patients with MPS. However, its efficacy can vary among individuals, and it is often used in combination with other therapies.

### 4.3. Conventional modalities

Various physical modalities are used to complement other therapies and provide symptomatic relief.

#### 4.3.1. Heat therapy

Applying heat to affected areas can help increase blood flow, relax muscles, and alleviate pain. Methods include hot packs, warm baths, and heating pads. Heat therapy is particularly beneficial for chronic muscle tension and stiffness

#### 4.3.2. Cold therapy

Cold packs or ice baths are used to reduce inflammation and numb pain in acute stages. This modality can be particularly useful after intense physical activity or when inflammation is present

#### 4.3.3. Ultrasound therapy

This modality uses high-frequency sound waves to promote tissue healing and reduce pain. It can help improve muscle flexibility and decrease inflammation in the affected muscles.

#### 4.3.4. Electrical stimulation

Techniques such as transcutaneous electrical nerve stimulation (TENS) use electrical impulses to modulate pain perception and reduce muscle spasms. TENS is commonly used as a supplementary treatment to provide pain relief.

Clinical treatment of MPS is primarily directed at the point of provocation, i.e., TPI, aims to interrupt the pain caused by MPS and correcting the structural and functional imbalance caused by myofascial trigger point formation.^[8]^ Trigger point needling remains 1 of the most popular and widely used methods today, and in general, TPI is often more effective than DN in reducing subjective pain scores and pain pressure threshold outcomes.^[9]^ Needle therapy is inexpensive and less invasive, and early clinical applications have shown good efficacy.

### 4.4. MPS injection treatment method

If the trigger point can be injected accurately, then a local twitch response or involvement of pain in the muscle will occur, and palpation by the physician alone to localize the provocation point often lacks objectivity and reliability; therefore, a guidance pathway that is free from subjective influences is needed for injection, and ultrasound (US) is the best choice. Niraj et al^[10]^ reported that the use of ultrasound guidance during injection makes the trigger point become more apparent in US imaging. In addition, studies have now recognized that US imaging during MTrP injection is clinically more effective for injection therapy, more likely to avoid needle misplacement, and thus prevent adverse outcomes for patients, low risk of US guidance, and improved accuracy of therapy (Table 1).^[11]^

**Table 1:** Comparison of advantages and disadvantages of various invasive and noninvasive treatment methods for MPS.

Treatment type	Treatment method	Advantages	Disadvantages	Reference studies
Invasive	Trigger point injections	– Immediate pain relief	– Risk of infection, bleeding, and nerve damage	Tontodonati M et al (2021). “Efficacy of trigger point injections in myofascial pain syndrome.” *Musculoskeletal Science and Practice*.
	Acupuncture	– Can reduce pain effectively	– Requires multiple sessions, practitioner-dependent	Lee JH et al (2020). “Effectiveness of acupuncture for myofascial pain syndrome.” *Pain Medicine*.
	Botulinum toxin injections	– Long-lasting pain relief	– High cost, potential side effects like muscle weakness	De Lemos D et al (2022). “Botulinum toxin for muscular and myofascial pain: A systematic review.” *Pain Physician*.
	Nerve blocks	– Targeted pain relief	– Invasive, risk of complications	Cohen SP et al (2020). “Nerve blocks for myofascial pain.” *Anesthesiology Clinics*.
NonInvasive	Exercise therapy	– Helpful in improving strength and functionality	– Requires patient adherence, possible exacerbation of pain	Goel V et al (2019). “Exercises for myofascial pain syndrome: A systematic review.” *Physical Therapy*.
	Manual therapy (e.g., massage)	– Reduces muscle tension and increases range of motion	– Effectiveness depends on therapist’s skill	Kottner J et al (2021). “The effect of manual therapy on myofascial pain.” *Clinical Rehabilitation*.
	Dry needling	– Targets trigger points, minimally invasive	– Temporary discomfort, may cause soreness	Cummings TM (2018). “Efficacy of dry needling for myofascial pain.” *Journal of Musculoskeletal Pain*.
	Ultrasound therapy	– noninvasive, may enhance healing	– Varies in effectiveness, not suitable for all conditions	Wang T et al (2018). “Ultrasound therapy in the treatment of myofascial pain syndrome.” *Clinical Rehabilitation*.

TPI was performed according to the steps described by Travell and Simons. When the patient was in the appropriate position, the location of the MTrP was determined under US guidance, marked with a pen, and the skin was disinfected with an appropriate antiseptic solution. For injection, the trigger point was stabilized between the thumb and index finger, and the needle was then inserted vertically into the skin and advanced until the trigger point location was reached, after ensuring that it was in negative suction, the drug was injected in small doses into the identified point. Patients who underwent TPI all showed a local muscle twitching response, demonstrating the correct injection location. The injection site was then compressed for approximately 2 minutes to ensure hemostasis.^[12,13]^

Injection of MTrP consists of 2 methods: the first method involves repeatedly stabbing the syringe needle into the MTrP, and once strong local soreness or swelling is caused, 0.1 to 0.2 mL of local anesthetic can be injected to relieve the pain, and the beneficial effect of this therapy is to reduce acute or chronic pain in the muscle; the second method is to add 0.3 to 0.5 mL of anesthetic and simply inject it into the muscle twitching location, followed by a gentle local massage to infiltrate the anesthetic into the MTrP site; this method avoids local tingling to a considerable extent, but its effect is short-lived compared to the former. Therefore, the treatment procedure of the second method must be repeated several times, and this method is suitable for some patients who are pain-sensitive and cannot tolerate the pain of needling.^[14]^ The appropriate injection method should be selected according to the patient’s condition and wishes when operating clinically.

### 4.5. Stimulated pain point injection therapy

The concept of “provoked pain point” was discovered and defined by Janet Travell and David Simons in the 1950s^[15]^ and has been studied for many years since then. MTrP can be divided into latent and active provoked pain points.^[4]^ The main clinical difference between the 2 is the patient’s recognition of the preexisting pain. Active provoked pain points are often considered to be associated with spontaneous and persistent pain, while latent provoked pain points are often ignored by patients because they are rarely spontaneous.^[6]^ For this reason, in addition to actively treating active provoked pain points, patients with MPS should focus on therapeutic management strategies for latent provoked pain points.

#### 4.5.1. Glucose

Prolotherapy, formally introduced by Dr George Hackett in the 1950s, has been used in clinical practice for more than 80 years to treat a variety of chronic musculoskeletal disorders by promoting the regeneration of damaged tissue and stimulating the recovery of wounds or degenerated tissue.^[16]^ The most commonly used clinical proliferative agent is glucose in concentrations ranging from 12.5% to 25% and sometimes contains additives such as zinc, human growth hormone, ozone, manganese, PRP, or bone marrow.^[17]^ Glucose is considered an ideal bulking agent (a natural sugar chemically identical to the body’s own glucose) because it is water soluble, a normal component of blood chemistry, and can be safely injected into multiple sites and used in large quantities. Injecting a glucose solution, dehydrates the cells at the injection site, causing local tissue trauma, which in turn attracts granulocytes and macrophages, promoting healing and relieving pain.^[17,18]^

In recent years, there have been an increasing number of trials using glucose as a point of MPS agonist injection, and the efficacy can be good. In a randomized controlled trial back in 1997, 3 groups of patients received injections of 5% glucose, saline, or 0.5% lidocaine, and 7 days after injection, patients treated with glucose showed improvement in pain at visual analog scale (VAS score) and pressure threshold tolerance compared to other interventions, and patients injected with saline and lidocaine showed no improvement in either index. Chou et al^[19]^ studied the efficacy of ultrasound-guided glucose injections in 45 patients with MPS who had failed to respond to other treatments. A statistical analysis of symptom severity before treatment and symptom response 1 month after treatment using visual analog scale (VAS) scores showed that 8 (24.4%) patients reported complete resolution of symptoms at the treatment site. Thirty-six (80.0%) patients reported more than 50% improvement in their symptoms. The mean VAS scores before and after treatment were 7.0 and 2.44 (*P* < .001), indicating an overall reduction in symptom severity of 65.0%. In a study of 67 patients with chronic MPS who received repeated injections of 15% glucose, the mean pain score decreased from 7.0 to 2.55, reaching statistical significance. A study of 177 patients with MPS who received 20% glucose injections showed significant relief of chronic myofascial pain and dysfunction, with more than 80% of patients returning to daily activities.

Although all regimens of glucose solutions have produced significantly positive results, studies comparing glucose with other treatment modalities have also emerged to determine who is superior in terms of efficacy, glucose vs placebo or other injectable regimens. However, because there is no consensus on the optimal regimen for glucose concentration, the glucose concentrations used in clinical studies are inconsistent, so further long-term research on this issue is needed in the future.

#### 4.5.2. Physiological saline

Clinically, the use of less active drugs in TPI is a beneficial option for patients with MPS, considering the possibility of various adverse effects associated with the application of other drugs, for example, TPI with sterile saline is a safe and cost-effective option for the treatment of MPS.

In 1955, Sola and Kuitert used normal saline (NS) injections to inactivate MTrP and found that saline was less risky than local anesthetics.^[20]^ In 1980 Frost et al^[21]^ found that 80% of patients in a double-blind controlled trial reported pain relief after saline injections; a study evaluating a combination of conventional active drugs vs NS alone in 48 MPS patients presenting to the emergency department The results of a trial evaluating the efficacy of TPI in 48 patients with MPS seen in the emergency department showed significant pain relief after TPI treatment in both groups, but TPI with NS may be preferred over conventional active medication because of its lower cost and fewer side effects, demonstrating that TPI plus NS is an effective first-line treatment for MPS.^[22]^ Although the analgesic mechanism of NS is not yet clear, clinical randomized trials have all shown its safety and efficacy in the treatment of MPS. The low cost, good safety profile, and reproducible, measurable analgesic quality suggest that the use of saline as a point of provocation injection offers a compelling treatment option for symptom control in MPS.

However, although most studies support the view that the nature of the injected substance makes no difference to the outcome, controversy remains, with some findings supporting the conclusion that active drugs are more effective than saline solutions or vice versa. In a double-blind study of 107 patients with head and neck fasciitis trigger points, divided into 3 groups and treated with local injections of 1 of 3 solutions (0.25% bupivacaine, 1% lidocaine, or 0.9% saline), no significant effect was produced in terms of pain control or overall treatment outcome.^[23]^ Future studies with larger sample sizes and better control of concomitant treatments may reaffirm the efficacy and safety of NS treatment with myofascial pain, and larger trials will also assess the generalizability of NS to MPS findings.

#### 4.5.3. Lidocaine

Local anesthetics are most commonly used to treat myofascial trigger points, and their primary use is to prevent local pain; commonly used anesthetics include lidocaine, bupivacaine, procaine, and mixtures of lidocaine and tretinoin. Studies have shown that TPI using local anesthetics can improve the degree, extent, and nociceptive pressure threshold of pain to some extent. The amount of local anesthetic injected has also been investigated, and small doses are considered the most effective, and typically <1 mL of a local anesthetic preparation should be injected in a highly controlled manner.^[24]^ Lidocaine is most commonly used clinically for TPI treatment.

Based on previous controlled studies, it was found that although the local anesthetic TPI did result in an improvement in patient pain, it was not a significantly better treatment. For example, Gandolfi et al^[25]^ found that collagen TPI was more effective than lidocaine, which in turn was more effective than saline TPI controls, Aksu et al^[26]^ found that DN and exercise were equally effective, and Roldan et al^[22]^ found that saline was as effective as local anesthetic TPI. Kamanli et al^[27]^ concluded that lidocaine and botulinum toxin type A (BTX-A) injections reduced pain and improved quality of life accordingly, although BTX-A injections were more effective and reduced patient anxiety and depression more than lidocaine, the authors still concluded that lidocaine was a better first-line treatment because it provided faster relief and is more cost-effective.

The literature suggests that the local anesthetic TPI often relieves pain in MPS and that other treatments, such as collagen or saline TPI, DN, and exercise, may also be useful first-line treatments.

#### 4.5.4. Botulinum toxin type A

Botulinum toxin type A (BTX-A) is a powerful neurotoxin synthesized by Clostridium botulinum that breaks the spasticity and pain cycle by blocking the release of acetylcholine into the neuromuscular junction, thereby causing a decrease in muscle contraction, since the 1990s, BTX-A has been used to treat a number of musculoskeletal disorders, including spasticity, cervical dystonia and blepharospasm. Through a combination of peripheral and central nervous system effects, BTX-A is thought to reduce the pain associated with MPS and may offer some advantages over current MPS treatment options. However, despite numerous clinical trials, the clinical efficacy of using BTX-A injections in relieving MPS remains controversial.

Göbel et al^[28]^ conducted a randomized, double-blind, placebo-controlled multicenter trial in 2006 that showed a statistically significant improvement in pain relief with BTX-A injections. This was the largest trial to date, with 145 patients enrolled. However, not all studies support the hypothesis that BTX-A improves MPS symptoms. Ferrante et al^[29]^ completed a randomized, controlled, double-blind study in 2005 that concluded, despite measures taken to control for various confounding factors that could affect the results, that no significant differences in pain were found between the placebo and BTX-A groups, and that direct injection of BTX-A into the trigger point did not improve myofascial pain. Other trials have shown no statistically significant differences between the BTX-A and placebo groups. However, in some trials, there was improvement in both the treatment and placebo groups, suggesting a significant placebo effect in these studies. Considering this effect, it is difficult to conclude that BTX-A injections are clinically ineffective for MPS. Based on the results of these trials, further studies in an effort to minimize the placebo effect are necessary to clarify the role of BTX-A in the treatment of MPS.

One possible explanation for the differing findings regarding the effectiveness of BTX-A injections for MPS is the wide variation in treatment duration prior to data collection, as has been suggested by previous reviews of the effectiveness of BTX-A injections.^[30]^ Another potential factor is the small sample size of many of the included studies. The sample sizes of the studies ranged from 18 to 153 patients, making it possible for any 1 study to be significantly biased or ineffective. The implementation of further clinical trials in the future should take into account issues such as minimizing placebo effects, repeated dosing, and adequate coverage of trigger points that could provide more conclusive evidence for BTX-A in the treatment of MPS.

#### 4.5.5. Platelet-rich plasma

Platelet-rich plasma (PRP) is a platelet concentrate extracted from autologous blood by centrifugation. The platelets in PRP are activated to release a variety of growth factors, such as platelet-derived growth factor (PDGF), insulin-like growth factor (IGF), vascular endothelial growth factor (VEGF), and epidermal growth factor (EGF), etc., which can effectively promote tissue cell regeneration and repair.^[31]^ In the late 1990s, surgeons began adding PRP to fibrin to form a gel to be placed at the surgical site with the goal of accelerating healing, promoting implant fixation, and speeding recovery. PRP is now used in orthopedics, cardiac surgery, plastic surgery, gynecology, urology, and other departments. Intramuscular injections of PRP promote the healing of painful muscles and have been used in the treatment of many diseases such as tendonitis, osteoarthritis, wound healing, and ophthalmic diseases.^[32]^ Many studies have shown that PRP has a better healing effect on muscle injuries and has potential benefits for the treatment of myofascial pain.

Several randomized controlled trials have evaluated the use of PRP in the treatment of chronic plantar MPS, and topical injections may be an alternative to plantar MPS to reduce concerns about adverse effects resulting from corticosteroid injections, such as the possibility of steroid injections in this area including muscle atrophy or plantar fascia rupture.^[33]^ A meta-analysis evaluated a controlled trial of PRP injections vs corticosteroid injections in terms of efficacy, and PRP was shown to be superior.^[34]^ Barrett^[35]^ reported that 78% of patients with plantar MPS treated with PRP had complete symptom relief within 1 year, according to the study. The credibility of this trial needs further validation due to its small sample size, low level of evidence, and the study’s lack of randomization and control groups. In addition to its application in the plantar myofascial, there are clinical trials of injections at facial trigger points. Yilmaz et al^[36]^ evaluated and compared the effectiveness of local anesthetics, botulinum toxin, and PRP injections on pain in patients with biting myofascial trigger points, and all 3 interventions were successful in improving symptoms of biting MTrP at 1 and 3 months. However, at 3-month follow-up, Botox injections were more superior and effective in controlling pain for up to 6 months. Similarly, Nitecka-Buchta^[32]^ et al found PRP injections to be very effective in reducing occlusal MTrP pain in the short term, possibly because the efficacy of PRP injections does not last long. According to previous studies, it has been shown that the best improvement is achieved 2 to 10 days after injection due to the platelet half-life of about 7 to 10 days.

Platelet-rich plasma is a therapeutic option that can be used alone or in combination with other methods, and the efficacy and safety of PRP have been demonstrated in many medical conditions. Although the effects of PRP have been extensively studied and its modified products are constantly being updated, its function and mechanism of action are not yet well understood, and further research on PRP is necessary. it is relatively certain that sterile preparations of PRP are clinically safe. However, due to irregularities in preparation methods and differing opinions on optimal blood component concentrations, it is difficult to determine its true efficacy, and in the future, more focus needs to be placed on developing standardized methods of PRP preparation and administration to confirm true efficacy. In addition, the current studies surrounding PRP treatment have significant limitations in terms of article bias, lack of standardization in the preparation process, and insufficient multicenter randomized large clinical trials, and in the future, more studies and long-term follow-up could be conducted to guide clinical practice even further.

#### 4.5.6. Platelet-poor plasma

Platelet-poor plasma (PPP) refers to a plasma product containing a small number of platelets after centrifugation and has long been considered a waste product left over from PRP and has not received much attention. Gradually, studies have reported that PPP is as effective as PRP in treating various musculoskeletal injuries.

Miroshnychenko et al^[37]^ showed that, unlike PRP, PPP reduced the proliferation rate of human primary skeletal muscle myogenic cells but significantly induced the differentiation of these cells towards the myogenic pathway and the formation of tendons, suggesting that the beneficial effects of PRP on muscle formation may be mainly due to the plasma itself. Indeed, although PPP by definition contains very low concentrations of platelets and therefore low amounts of growth factors, it is still a reservoir of biologically active molecules (e.g. PDGF, IGF-1). Although PPP may hold promise for skeletal muscle injury, further studies are needed to evaluate the exact growth factors contained in this plasma preparation to reveal its role in skeletal muscle tissue regeneration in vivo.^[38]^ A recent in vivo study surprisingly showed that PPP has an earlier angiogenic effect compared to PRP.^[39]^ Various pilot studies in different musculoskeletal regions have shown that PPP injections have a similar biological response to PRP and may have a positive effect on degenerative or wound tissue remodeling.^[37,40–43]^ A clinical trial comparing PRP and PPP injections in patients with chronic persistent plantar fasciitis showed significant improvement in pain in these patients with both treatment regimens at a 6-month follow-up after injection.^[44]^ This finding questions the superiority of PRP injections.

At present, the treatment of MPS by PPP injection is still in its infancy, and there is no uniformity in its preparation and operational standards; therefore, further randomized controlled trials with long-term follow-up are needed to produce definitive MPS efficacy results.

#### 4.5.7. Steroids

The use of intra-articular injections of corticosteroids in the treatment of inflammatory arthritis and osteoarthritis has been well established, and some studies also support the use of corticosteroid injections for the treatment of fasciitis, usually routinely with corticosteroid preparations such as methylprednisolone, tretinoin, betamethasone alone or in combination with local anesthetics for pain relief and local anti-inflammatory treatment of various joint and soft tissue conditions.^[45]^ Local anesthetics added to corticosteroid preparations to provide immediate short-term pain relief may facilitate effective diffusion of the injections or provide diagnostic feedback by differentiating whether the pain is local or referred to.^[46]^ There is no conclusive evidence as to whether steroid injections are more effective than other drugs in the treatment of MPS.

In an early high-quality foreign study, Garvey et al^[47]^ compared TPIs of corticosteroids with placebo in 63 patients with lumbar fasciitis pain who were able to palpate the provocative pain point, with no significant difference in the improvement in lumbar pain reported by the 2 groups after 2 weeks. Also, in an early foreign study, Sonne et al^[48]^ studied the effects of local iliolumbar ligament injections of corticosteroids and placebo in 30 patients and found no significant difference in pain relief at 2 weeks, but follow-up after 2 weeks found significantly higher pain improvement in the corticosteroid group than in the placebo group. In China, Zhang Yizen et al^[49]^ compared the effect of sodium hyaluronate with corticosteroids in the treatment of plantar fasciitis in 65 patients. After 1 month of treatment, the patients in the corticosteroid group had significantly less pain than those in the sodium hyaluronate group, demonstrating the positive short-term effect of corticosteroids in relieving pain. Raissi et al^[50]^ compared the effect of dextrose prolotherapy with corticosteroids injection in the treatment of plantar fasciitis and concluded that glucose injection treatment was slower in onset and less effective than steroidal injection in the long term.

Glucocorticoids have been widely used in the treatment of fasciitis, which is simple, fast-acting, and inexpensive, with the disadvantage that the duration of efficacy is short, and the frequent use of these drugs is associated with adverse effects of systemic effects and local atrophy and rare but serious complications of steroid injections have been mentioned in some of the literature.^[51]^ Facial flushing is a common side effect of corticosteroid injections, with a reported incidence of 10% to 15%, based on previous findings that flushing occurs more frequently with tretinoin and are dose-related, and that side effects occurring 36 hours after corticosteroid injection are usually self-limiting. In this country, to avoid the risk of injections, fewer trials will choose steroid preparations for the treatment of MPS.

## 5. Conclusion

As a commonly used clinical treatment for MPS, TPI has the advantages of low cost, low trauma, good efficacy, and clear targeting, and has been the treatment of choice for clinicians for many years, playing a very important role in relieving patients’ pain and improving their quality of life. The drugs injected at the point of excitation mainly include hypertonic glucose, saline, lidocaine, botulinum toxin type A, PRP, PPP, steroids, etc., which can provide rapid pain relief in the short term, but often require patients to overcome pain and other symptoms, and the long-term efficacy is yet to be verified.

## 6. Limitations

Analysis of a large amount of domestic and international literature shows that there are still some problems in the research process of radical pain point injection therapy for MPS: The number of high-quality randomized controlled trials is limited, and there are problems such as small sample size, unclear randomization, and short follow-up time; there are no uniform standards for the dose of individual agonist injections, how many agonists are injected in a single treatment, and the frequency of injection; drug safety issues, local anesthetics can cause damage to local skeletal muscle in excessive doses, and the preparation of autologous plasma preparations may be contaminated during the process, leading to the occurrence of local tissue infections, and long-term glucocorticoid injections can lead to side effects such as osteoporosis, muscle atrophy, hirsutism, and skin thinning.

## Author contributions

**Conceptualization:** Nadia Anwar, Yuan Jie.

**Project administration:** Nadia Anwar.

**Resources:** Nadia Anwar, Hao Jin.

**Supervision:** Zhaoqiong Zhu.

**Validation:** Nadia Anwar, Xiong Wei.

**Visualization:** Nadia Anwar, Zhao Hongbo.

**Writing – original draft:** Nadia Anwar.

**Writing – review & editing:** Nadia Anwar.

## References

[1] Zhang Q Fu C Huang L. Efficacy of extracorporeal shockwave therapy on pain and function in myofascial pain syndrome of the trapezius: a systematic review and meta-analysis. Arch Phys Med Rehabil. 2020;101:1437–46. 32234411 10.1016/j.apmr.2020.02.013

[2] Kasper D Fauci A Hauser S Longo D Jameson J Loscalzo J. Harrison’s Principles of Internal Medicine. 19th ed. New York: Mcgraw-hill; 2015.

[3] Fricton J. Myofascial pain: mechanisms to management. Oral Maxillofac Surg Clin North Am. 2016;28:289–311. 27475508 10.1016/j.coms.2016.03.010

[4] Simons DG. Travell and Simons’: Myofascial Pain and Dysfunction The Trigger Point Manual, Volume 1: Upper Half of Body, Second Edition, Regional Pain and Anesthesia Medicine: Lippincott Williams & Wilkins, 1999:11–93.

[5] Fernández-de-Las-Peñas C Dommerholt J. International consensus on diagnostic criteria and clinical considerations of myofascial trigger points: a Delphi study. Pain Med. 2018;19:142–50. 29025044 10.1093/pm/pnx207

[6] Shah JP Thaker N Heimur J Aredo JV Sikdar S Gerber L. Myofascial trigger points then and now: a historical and scientific perspective. PM R. 2015;7:746–61. 25724849 10.1016/j.pmrj.2015.01.024 PMC4508225

[7] Hsieh L-F Hong C-Z Chern S-H Chen C-C. Efficacy and side effects of diclofenac patch in treatment of patients with myofascial pain syndrome of the upper trapezius. J Pain Symptom Manage. 2010;39:116–25. 19822404 10.1016/j.jpainsymman.2009.05.016

[8] Borg-Stein J Iaccarino MA. Myofascial pain syndrome treatments. Phys Med Rehabil Clin N Am. 2014;25:357–74. 24787338 10.1016/j.pmr.2014.01.012

[9] Srbely JZ. New trends in the treatment and management of myofascial pain syndrome. Curr Pain Headache Rep. 2010;14:346–52. 20607458 10.1007/s11916-010-0128-4

[10] Niraj G Collett B Bone M. Ultrasound-guided trigger point injection: first description of changes visible on ultrasound scanning in the muscle containing the trigger point. Br J Anaesth. 2011;107:474–5. 10.1093/bja/aer247 21841058

[11] Kumbhare D Singh D Rathbone A. Ultrasound-guided interventional procedures: myofascial trigger points with structured literature review. Reg Anesth Pain Med. 2017;42:407–12. 28277418 10.1097/AAP.0000000000000572

[12] Simons DG. Diagnostic criteria of myofascial pain caused by trigger points. J Musculoskelet Pain. 1999;7:111–20.

[13] Barbero M Schneebeli A Koetsier E Maino P. Myofascial pain syndrome and trigger points: evaluation and treatment in patients with musculoskeletal pain. Curr Opin Support Palliat Care. 2019;13:270–6. 31313700 10.1097/SPC.0000000000000445

[14] Zhuang X Tan S Huang Q. Understanding of myofascial trigger points. Chin Med J (Engl). 2014;127:4271–7. 25533832

[15] Travell JG Simons DG. Myofascial Pain and Dysfunction: the Trigger Point Manual. USA: Lippincott Williams & Wilkins; 1992.

[16] Hackett G Hemwall G Montgomery G. Ligament and Tendon Relaxation Treated by Prolotherapy. Springfield, IL: Charles C. Thomas Publisher; 1993.

[17] Money S. Pathophysiology of trigger points in myofascial pain syndrome. J Pain Palliat Care Pharmacother. 2017;31:158–9. 28379050 10.1080/15360288.2017.1298688

[18] Cole B Lam P Hackett L Murrell GA. Ultrasound-guided injections for supraspinatus tendinopathy: corticosteroid versus glucose prolotherapy–a randomized controlled clinical trial. Shoulder Elbow. 2018;10:170–8. 29796104 10.1177/1758573217708199 PMC5960868

[19] Chou Y Chiou H-J Wang H-K Lai Y-C. Ultrasound-guided dextrose injection treatment for chronic myofascial pain syndrome: a retrospective case series. J Chin Med Assoc. 2020;83:876–9. 32349034 10.1097/JCMA.0000000000000339 PMC7478217

[20] Sola AE Kuitert JH. Myofascial trigger point pain in the neck and shoulder girdle; report of 100 cases treated by injection of normal saline. Northwest Med. 1955;54:980–4. 13253967

[21] Frost F Jessen B Siggaard-Andersen J. Myofascial pain treated with injections. A controlled double-blind trial. Ugeskr Laeger. 1980;142:1754–7. 7008300

[22] Roldan CJ Osuagwu U Cardenas-Turanzas M Huh BK. Normal saline trigger point injections vs conventional active drug mix for myofascial pain syndromes. Am J Emerg Med. 2020;38:311–6. 31477359 10.1016/j.ajem.2019.158410

[23] Tschopp KP Gysin C. Local injection therapy in 107 patients with myofascial pain syndrome of the head and neck. ORL J Otorhinolaryngol Relat Spec. 1996;58:306–10. 8958538 10.1159/000276860

[24] Lavelle ED Lavelle W Smith HS. Myofascial trigger points. Anesthesiol Clin. 2007;25:841–51, vii. 18054148 10.1016/j.anclin.2007.07.003

[25] Ga H Koh H-J Choi J-H Kim C-H. Intramuscular and nerve root stimulation vs lidocaine injection to trigger points in myofascial pain syndrome. J Rehabil Med. 2007;39:374–8. 17549328 10.2340/16501977-0058

[26] Aksu O Doğan YP Çağlar NS Şener BM. Comparison of the efficacy of dry needling and trigger point injections with exercise in temporomandibular myofascial pain treatment. Turk J Phys Med Rehabil. 2019;65:228. 31663071 10.5606/tftrd.2019.1802 PMC6797918

[27] Kamanli A Kaya A Ardicoglu O Ozgocmen S Zengin FO Bayik Y. Comparison of lidocaine injection, botulinum toxin injection, and dry needling to trigger points in myofascial pain syndrome. Rheumatol Int. 2005;25:604–11. 15372199 10.1007/s00296-004-0485-6

[28] Göbel H Heinze A Reichel G Hefter H Benecke R; Dysport myofascial pain study group. Efficacy and safety of a single botulinum type A toxin complex treatment (Dysport®) for the relief of upper back myofascial pain syndrome: results from a randomized double-blind placebo-controlled multicentre study. Pain. 2006;125:82–8. 16750294 10.1016/j.pain.2006.05.001

[29] Ferrante FM Bearn L Rothrock R King L. Evidence against trigger point injection technique for the treatment of cervicothoracic myofascial pain with botulinum toxin type A. Anesthesiology. 2005;103:377–83. 16052120 10.1097/00000542-200508000-00021

[30] Khalifeh M Mehta K Varguise N Suarez-Durall P Enciso R. Botulinum toxin type A for the treatment of head and neck chronic myofascial pain syndrome: a systematic review and meta-analysis. J Am Dent Assoc. 2016;147:959–73.e1. 27737756 10.1016/j.adaj.2016.08.022

[31] El-Sharkawy H Kantarci A Deady J. Platelet-rich plasma: growth factors and pro-and anti-inflammatory properties. J Periodontol. 2007;78:661–9. 17397313 10.1902/jop.2007.060302

[32] Nitecka-Buchta A Walczynska-Dragon K Kempa WM Baron S. Platelet-rich plasma intramuscular injections—antinociceptive therapy in myofascial pain within masseter muscles in temporomandibular disorders patients: a pilot study. Front Neurol. 2019;10:250. 30941095 10.3389/fneur.2019.00250 PMC6433706

[33] Gupta S Paliczak A Delgado D. Evidence-based indications of platelet-rich plasma therapy. Expert Rev Hematol. 2021;14:97–108. 33275468 10.1080/17474086.2021.1860002

[34] Jain K Murphy PN Clough TM. Platelet rich plasma versus corticosteroid injection for plantar fasciitis: a comparative study. Foot (Edinb). 2015;25:235–7. 26362235 10.1016/j.foot.2015.08.006

[35] Barrett SL Erredge SE. Growth factors for chronic plantar fasciitis? Podiatry Today. 2004;17:37.

[36] Yilmaz O Sivrikaya EC Taskesen F Pirpir C Ciftci S. Comparison of the efficacy of botulinum toxin, local anesthesia, and platelet-rich plasma injections in patients with myofascial trigger points in the masseter muscle. J Oral Maxillofac Surg. 2021;79:88.e1–9. 10.1016/j.joms.2020.09.013 33045182

[37] Miroshnychenko O Chang WT Dragoo JL. The use of platelet-rich and platelet-poor plasma to enhance differentiation of skeletal myoblasts: implications for the use of autologous blood products for muscle regeneration. Am J Sports Med. 2017;45:945–53. 28027451 10.1177/0363546516677547

[38] Chellini F Tani A Zecchi-Orlandini S Sassoli C. Influence of platelet-rich and platelet-poor plasma on endogenous mechanisms of skeletal muscle repair/regeneration. Int J Mol Sci. 2019;20:683. 30764506 10.3390/ijms20030683 PMC6387315

[39] Shahidi M Vatanmakanian M Arami MK. A comparative study between platelet-rich plasma and platelet-poor plasma effects on angiogenesis. Med Mol Morphol. 2018;51:21–31. 28948378 10.1007/s00795-017-0168-5

[40] Yilmaz S Kabadayi C Ipci SD Cakar G Kuru B. Treatment of intrabony periodontal defects with platelet-rich plasma versus platelet-poor plasma combined with a bovine-derived xenograft: a controlled clinical trial. J Periodontol. 2011;82:837–44. 21138357 10.1902/jop.2010.100503

[41] Hatakeyama I Marukawa E Takahashi Y Omura K. Effects of platelet-poor plasma, platelet-rich plasma, and platelet-rich fibrin on healing of extraction sockets with buccal dehiscence in dogs. Tissue Eng Part A. 2014;20:874–82. 24098948 10.1089/ten.tea.2013.0058 PMC3926147

[42] Cáceres M Martínez C Martínez J Smith PC. Effects of platelet-rich and-poor plasma on the reparative response of gingival fibroblasts. Clin Oral Implants Res. 2012;23:1104–11. 22092788 10.1111/j.1600-0501.2011.02274.x

[43] Martínez CE González SA Palma V Smith PC. Platelet-poor and platelet-rich plasma stimulate bone lineage differentiation in periodontal ligament stem cells. J Periodontol. 2016;87:e18–26. 26367495 10.1902/jop.2015.150360

[44] Malahias M-A Mavrogenis AF Nikolaou VS. Similar effect of ultrasound-guided platelet-rich plasma versus platelet-poor plasma injections for chronic plantar fasciitis. Foot (Edinb). 2019;38:30–3. 30572281 10.1016/j.foot.2018.11.003

[45] MacMahon PJ Eustace SJ Kavanagh EC. Injectable corticosteroid and local anesthetic preparations: a review for radiologists. Radiology. 2009;252:647–61. 19717750 10.1148/radiol.2523081929

[46] Deshmukh AJ Thakur RR Goyal A Klein DA Ranawat AS Rodriguez JA. Accuracy of diagnostic injection in differentiating source of atypical hip pain. J Arthroplasty. 2010;25:129–33. 20570105 10.1016/j.arth.2010.04.015

[47] Garvey TA Marks MR Wiesel SW. A prospective, randomized, double-blind evaluation of trigger-point injection therapy for low-back pain. Spine. 1989;14:962–4. 2528826 10.1097/00007632-198909000-00008

[48] Sonne M Christensen K Hansen SE Jensen EM. Injection of steroids and local anaesthetics as therapy for low-back pain. Scand J Rheumatol. 1985;14:343–5. 2934803 10.3109/03009748509102036

[49] Zhang Y Wang P Liu Z. Ultrasound-guided injection of hyaluronic acid and corticosteroid for treating plantar fasciitis: evaluation of pain, fascia thickness and ankle-foot function. Chin J Tissue Eng Res. 2021;25:1670.

[50] Raissi G Arbabi A Rafiei M. Ultrasound-guided injection of dextrose versus corticosteroid in chronic plantar fasciitis management: a randomized, double-blind clinical trial. Foot Ankle Spec. 2023;16:9–19. 33461323 10.1177/1938640020980924

[51] Staal JB De Bie RA De Vet HC Hildebrandt J Nelemans P. Injection therapy for subacute and chronic low back pain: an updated Cochrane review. Spine. 2009;34:49–59. 19127161 10.1097/BRS.0b013e3181909558

